# Supplementary material for: Shrinking Bouma’s window: How to model crowding in dense displays
Source: PLoS Comput Biol. 2021 Jul 6;17(7):e1009187. doi: 10.1371/journal.pcbi.1009187 (PMC8284675; doi:10.1371/journal.pcbi.1009187)
Supplement: S1 Appendix — Detailed description of the model, more details about the results. (PDF) [file pcbi.1009187.s001.pdf]

S1 Appendix: Bouma's law model

To set a basis for our analysis, we used a model that assumes Bouma's law (1) holds true in dense displays. In this model (Fig A, top), any flanker in the dense display creates the same amount of interference as it would do in a sparse display. To set interaction weights between the flankers and the target, we used the data from the sparse display experiment of Van der Burg et al. (1; Fig 2a in main text, bottom). Based on this data, we defined interaction weights for any flanker as the performance drop that it would cause in the sparse display experiment, and the total interaction T as the sum of the weights of all flankers in the display. For each display, we defined the probability for the model to make a correct response as in Eq. 1.

$$P_{correct} = max\left[P_{unflanked} \cdot (1 - A \cdot T), 0.5\right] \quad (1)$$

$P_{unflanked}$ comes from the sparse display experiment in Van der Burg et al. (2) and is the average proportion of correct responses without flankers and A is a global gain for the interaction weights. A was set to 1.0 for sparse displays but was lowered to 0.3 for dense displays to avoid the model being always at chance level. It was tuned to obtain approximately 67% performance for the first generation in the GA procedure. Performance for each display was defined as the probability of correct responses.

Note that this model was used in Van der Burg et al. (2), to investigate whether the GA procedure was able to produce behaviour consistent with Bouma's law in the first place. However, directly using the probability of correct responses to select the best displays at each generation, without simulating trials, might have discounted variability in the evolution process of the GA. Hence, for completeness, we ran a second version of the model that, instead, selected the best displays based on the simulation of 12 trials (still using the probability of

correct responses as in Eq. 1, the first version of the model corresponds to running the second one with an infinite number of trials).

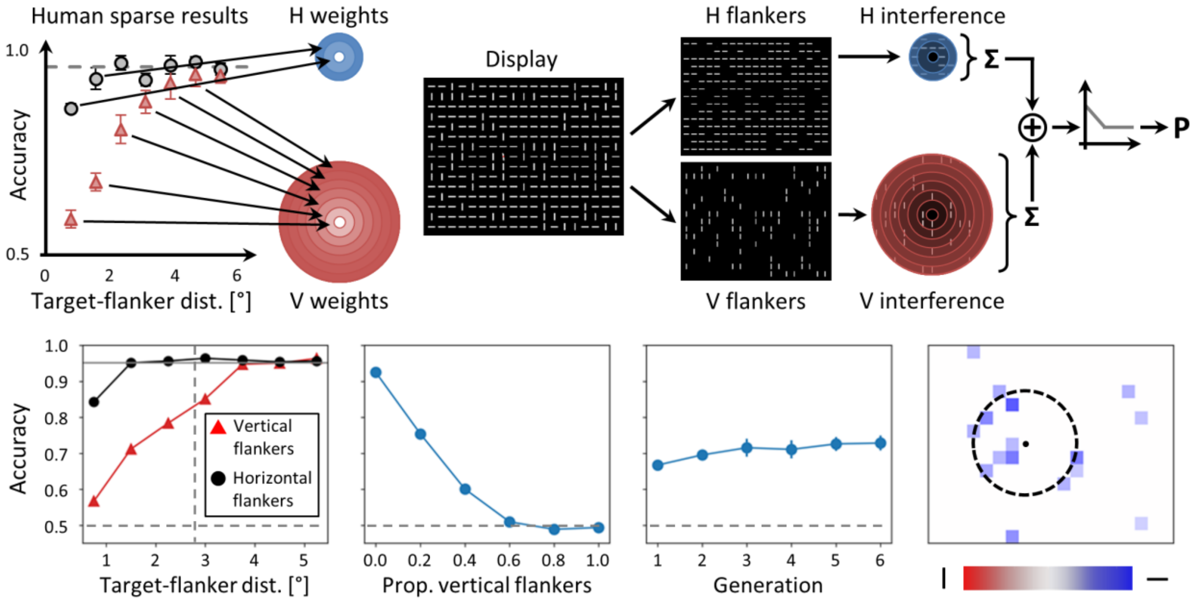

Fig A. Top. Bouma model. Flanker-target distance-dependent weights were defined as how much performance dropped from the unflanked level in the sparse display experiment of Van der Burg et al. (2). The input of the model is an array of 15 by 19 bits that encodes each flanker orientation. Spatial units in the model are implicitly encoded by setting the weights of each flanker location to the human data. For each display, the probability of correct response is a decreasing function of the sum of its flankers' weights (see Eq. 1). **Bottom.** Results obtained with the second version of the Bouma model (same description as in Fig 3 in the main text).

The results for both ways of selecting the best displays between generations are shown in Fig 3 in the main text (2nd row) for the first version and in Fig A (bottom) for the second version. Both versions reproduced human results for the sparse display and the proportion measures. Model performance improved as much as in the human experiment during the GA procedure for the first version, but the second version produced only a minor improvement. This may be due to the variability added by the selection process in the second version of the model. In consequence, the GA procedure did not highlight any specific location in the selection measure for the second version of the model, whereas essentially all elements inside Bouma's window

were highlighted for the first version. In summary, both versions of the model did not account for the shrinking of Bouma's window.

References

1. Bouma H. Visual interference in the parafoveal recognition of initial and final letters of words. Vision Res. 1973;13(4):767-82.

2. Van der Burg E, Olivers CN, Cass J. Evolving the keys to visual crowding. J Exp Psychol Hum Percept Perform. 2017;43(4):690.
