# Supplementary material for: Shrinking Bouma’s window: How to model crowding in dense displays
Source: PLoS Comput Biol. 2021 Jul 6;17(7):e1009187. doi: 10.1371/journal.pcbi.1009187 (PMC8284675; doi:10.1371/journal.pcbi.1009187)
Supplement: S2 Appendix — Detailed description of the model. (PDF) [file pcbi.1009187.s002.pdf]

S2 Appendix: Population coding model

The population coding model (1) provides a physiologically plausible description of the spatial integration of orientation signals and accounts for various aspects of visual crowding. In this model, a population of orientation-sensitive neurons encodes the content of each location in the stimulus array (Fig A). These neuron populations constitute the first layer of the model. Neurons in the second layer pool stimulus information locally, using a weighted summation of the population activities in the first layer. The weighting fields are expressed in cortical coordinates and hence depend on the population eccentricity. Then, orientation is decoded from the activity of the population in the second layer that corresponds to the target location. A mixture of von Mises distribution is fit to the population activity and the maximum value of the fitted function is taken as the decoded orientation. For each display, performance was computed as the proportion of decoded orientations of same sign as the target orientation.

Model parameters were the same as in (1), except for the pooling range that was adapted to produce Bouma's law in sparse displays. For dense displays, the model was very close to chance level, because the pooled activity from horizontal flankers was so large that it overwhelmed the activity coming directly from the target. To solve this issue, we added a prior to select target orientation: the value of the fitted von Mises mixture function was set to zero for any orientation outside the range [-45°, 45°], before it was used to decode the target orientation. However, even using the former prior, simply because there were too many flankers that were pooled in dense displays, the model was too close to chance level for the GA to work (performance could not increase during the GA procedure). To help the model reaching 67% of accuracy in the first generation, we increased the target orientation to ±10° (instead of ±5°), for dense displays only.

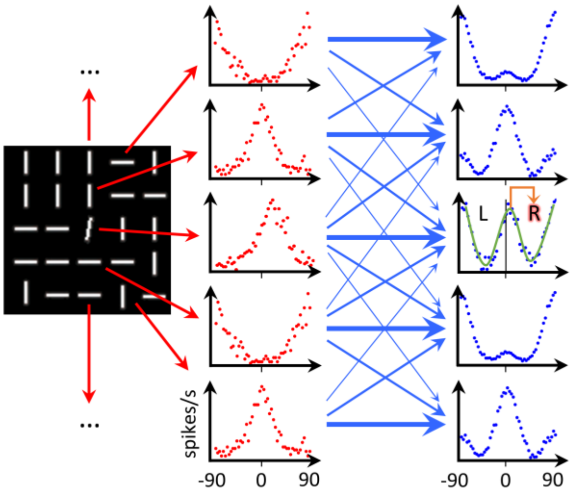

Fig A. Population coding model. Populations of orientation-selective neurons encode the content of every element in the display array (red arrows). The input of the model is an array of 15 by 19 bits that encodes each flanker orientation (only a subset is shown here). Then, the activity responsible for each location of the array is pooled to a second layer of neuron populations (blue arrows). Pooling weights (represented here by the thickness of the arrows) depend on the cortical distances between the populations. Am important parameter of the model is the cortical pooling range (which defines spatial units in the model) and was fitted to yield Bouma's law in sparse displays. Finally, the target orientation is decoded from the second layer activity by fitting a mixture of von Mises distributions (green) to the activity of the population responsible for the target. The sign of the target orientation is used to report a left or a right target.

Results obtained with the model are shown in Fig 3 in the main text (3rd row). The model reproduced human behaviour very well for the sparse display measure. For the proportion measure, the model performed better than humans for small proportions of vertical flankers. This may have been due to the prior that we added to the decoding process of the model. The GA procedure increased model performance dramatically, even to a larger extent than in the human experiment. The selection measure highlighted a large portion of the locations inside Bouma's window, which is not in accordance with the human results. Note that there was an inward-outward anisotropy (2) in the highlighted locations, i.e., flankers on the peripheral side of the target had more impact than flankers on the foveal side. This can be explained, because

the model takes cortical magnification into account: pooling distances are expressed in cortical units and hence, pooling has a larger range for populations located in the periphery than near the fovea. In summary, this model reproduces human results for all measures, except the selection measure.

References

1. Van den Berg R, Roerdink JB, Cornelissen FW. A neurophysiologically plausible population code model for feature integration explains visual crowding. PLoS Comput Biol. 2010;6(1):e1000646.

2. Toet A, Levi DM. The two-dimensional shape of spatial interaction zones in the parafovea. Vision Res. 1992;32(7):1349-57.
