# Supplementary material for: Shrinking Bouma’s window: How to model crowding in dense displays
Source: PLoS Comput Biol. 2021 Jul 6;17(7):e1009187. doi: 10.1371/journal.pcbi.1009187 (PMC8284675; doi:10.1371/journal.pcbi.1009187)
Supplement: S3 Appendix — Detailed description of the model. (PDF) [file pcbi.1009187.s003.pdf]

S3 Appendix: Texture model

Texture models (1) iteratively update an array of pure noise, until an image is produced that matches a specific set of statistics computed from the model's visual input. These models are seen as models of vision, because they provide a very efficient way to encode visual information in the brain, even for natural images (which are rather complex in terms of visual content, like our dense displays). Balas et al. (2) proposed that crowding is the result of such statistics being computed over pooling regions. They proposed to use the model of Portilla et Simoncelli (1) over a Bouma-sized patch centred on the target to generate textures whose content reflect the amount of crowding associated to the flanker pattern present in the input image. Rosenholtz et al. (3) proposed to improve this model by computing the statistics over many tiled regions whose size grow with eccentricity. However, we did not use the latter model because it was computationally too heavy: given the stimulus dimensions, it would have taken approximately 2 years to run the GA procedure on our lab computer.

We used the code available at https://github.com/LabForComputationalVision/textureSynth to produce the same kind of Bouma-sized textures, using the displays of Van der Burg et al. (4). For each display trial, we generated a texture and decoded whether the target was oriented to the left or to the right, using a template match algorithm (Fig Aa). The algorithm uses left and right target templates and looks for the best match over the whole texture. Every trial produced a different texture image, because the generative process is stochastic. The performance of the model was then the fraction of correct responses over the trials.

The reason why an algorithm was used instead of human observers looking at the textures, (as in 40) is that, to create new generations of displays, the GA procedure must know the

performance associated to the parent displays. Hence, it would have required the textures to be generated during the experiment, which would have added about 1 minute of texture computation between every button press in a human experiment, making it last about 64 hours per human participant. To make sure that our template match algorithm captured human performance qualitatively, we ran an experiment in which humans looked freely at the Bouma-sized textures generated by the model for dense displays in which the proportion of vertical flankers was varied. The task was to decide whether the texture came from a display that contained a target tilted to the left or to the right compared to vertical. We fitted the parameters of the template match algorithm to match human performance (Fig Ab).

As with the population coding model, the results of the texture model for dense displays were too close to chance level. Therefore, we increased the target orientation to ±15° (instead of ±5°), for dense displays only, so that performance was around 67% for the first generation of displays in the GA procedure. Note that this was not the case with the validation experiment we ran to produce the panel in Fig Ab.

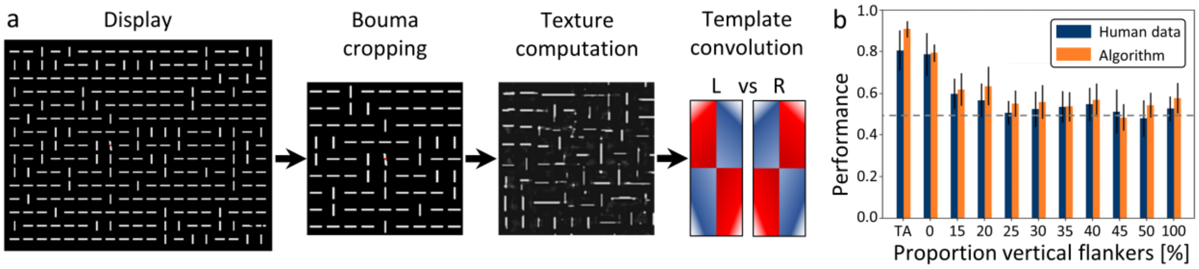

Fig A. **a.** Texture model. The input to the model is an actual image of the visual stimulus. Spatial units in the model are defined by the resolution of the stimulus, which was set to 30 pixels per degree. First, the stimulus display is cropped, so that only a Bouma-sized patch around the target is sent to the texture model. Then, the model iteratively matches a set of statistics between the input patch and the output texture. Finally, an algorithm chooses whether the texture comes from a display in which the target is tilted to the left or to the right by convolving left and right filters to the output texture and looking for the maximal match. **b.** Comparison between the template

match algorithm and experimental results in which human observers discriminated the target orientation from the output textures in free-viewing conditions, for different proportions of vertical flankers (TA stands for target alone). The algorithm captures human behaviour.

The results obtained with the model are shown in Fig 3 in the main text (4th row). For the sparse display measure, the model performance did not show a clear dependence on target-flanker distance, aside from the performance bump that happened when the flankers went outside the cropping range. This suggests that interference in this model does not depend on the relative location of elements, which is in contradiction with human results. This was already a hint that the model would not highlight special configuration in the GA procedure but would at best behave like the second version of the Bouma model. As expected, although the model reproduced human results for the proportion measure, performance did not improve in the GA procedure and the selection measure did not highlight any location, exactly as with the second version of the Bouma model (Fig A in S1 Appendix, bottom). In summary, the texture model only reproduced human results for the proportion measure.

References

1. Portilla J, Simoncelli EP. A parametric texture model based on joint statistics of complex wavelet coefficients. Int J Comput Vis. 2000;40(1):49-70.

2. Balas B, Nakano L, Rosenholtz R. A summary-statistic representation in peripheral vision explains visual crowding. J Vis. 2009;9(12):13-13.

3. Rosenholtz R, Yu D, Keshvari S. Challenges to pooling models of crowding: Implications for visual mechanisms. J Vis. 2019;19(7):15-15.

4. Van der Burg E, Olivers CN, Cass J. Evolving the keys to visual crowding. J Exp Psychol Hum Percept Perform. 2017;43(4):690.
