# Supplementary material for: Shrinking Bouma’s window: How to model crowding in dense displays
Source: PLoS Comput Biol. 2021 Jul 6;17(7):e1009187. doi: 10.1371/journal.pcbi.1009187 (PMC8284675; doi:10.1371/journal.pcbi.1009187)
Supplement: S4 Appendix — Detailed description of the model. (PDF) [file pcbi.1009187.s004.pdf]

S4 Appendix: CNN classifier

Deep feedforward convolutional neural networks (CNNs) share many similarities with humans in their architecture, in their activity patterns (1,2), as well as in the performance they reach in a large number of visual tasks (3,4). Here, we used the same method as in (5), testing AlexNet (6) as a representative of CNNs, because it is often used as a model of the human visual system (7–10). The weights of AlexNet were already trained on ImageNet (11). To perform the crowding task, we trained different classifiers to decode target orientation (left or right) based on the activity of each layer of the network. The training set was made of images that contained both the target and an array of vertical and horizontal flankers (Fig A). Only the weights of the classifier were affected by the training phase. In the image samples of the training set, the target never overlapped with the flanker array. After this training phase, the model used in the GA procedure consisted in AlexNet, plus the classifier whose layer gave the best fit of Bouma's law for sparse displays (which was the fourth layer). The performance of the model was then simply the fraction of correct classifications over the trials.

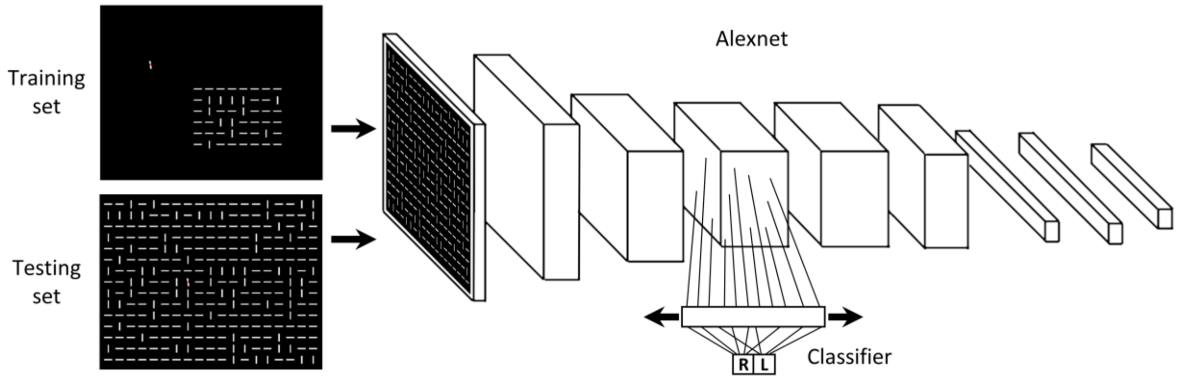

Fig A. CNN classifier. The input to the model is an actual image of the visual stimulus. Spatial units in the model are defined by the resolution of the stimulus, which was set to 15 pixels per degree, i.e., the maximum value given the input shape that AlexNet accepts (227 by 227 pixels). The stimulus display is processed by the architecture of Alexnet. On top of each layer, a decoder was trained to discriminate between left or right targets from the layer

activity. The weights of Alexnet (which have been previously trained on ImageNet) did not change during the training process. The training set was composed of samples containing the target alone and an array of vertical and horizontal flankers that never overlapped with the target. The loss function of the classifier was the cross-entropy on target classification. After training the classifiers, the whole model was tested with the four measures described in the Methods section. The reported results came from the trained classifier put on top of the layer that gave the best fit of Bouma's law in the sparse display measure. Adapted with permission from (5).

The results obtained with the model are shown in Fig 3 in the main text (5th row). None of the layers reproduced Bouma's law qualitatively in the sparse display measure. We report all measures that we obtained with a classifier put on top of the fourth layer of Alexnet, which gave the least bad fit, and whose receptive field size roughly matches the size of Bouma's window. For dense displays, the model performance generally decreased with the proportion of vertical flankers. However, the model was at chance level with 100% of horizontal flankers. During the GA procedure, model performance increased only marginally. The selection measure did not highlight any specific location that was crucial for this improvement. In summary, the CNN classifier replicated none of the human results.

References

1. Lindsey J, Ocko SA, Ganguli S, Deny S. A unified theory of early visual representations from retina to cortex through anatomically constrained deep CNNs. ArXiv Prepr ArXiv190100945. 2019;

2. Zeiler MD, Fergus R. Visualizing and understanding convolutional networks. In: European conference on computer vision. Springer; 2014. p. 818-33.

3. Eslami SA, Rezende DJ, Besse F, Viola F, Morcos AS, Garnelo M, et al. Neural scene representation and rendering. Science. 2018;360(6394):1204-10.

4. Girshick R, Radosavovic I, Gkioxari G, Dollár P, He K. Detectron. 2018.

5. Doerig A, Bornet A, Choung OH, Herzog MH. Crowding reveals fundamental differences in local vs. global processing in humans and machines. Vision Res. 2020;167:39-45.

6. Krizhevsky A, Sutskever I, Hinton GE. Imagenet classification with deep convolutional neural networks. In: Advances in neural information processing systems. 2012. p. 1097-105.

7. Khaligh-Razavi S-M, Kriegeskorte N. Deep supervised, but not unsupervised, models may explain IT cortical representation. PLoS Comput Biol. 2014;10(11):e1003915.

8. Kietzmann TC, McClure P, Kriegeskorte N. Deep neural networks in computational neuroscience. BioRxiv. 2018;133504.

9. VanRullen R. Perception science in the age of deep neural networks. Front Psychol. 2017;8:142.

10. Yamins DL, Hong H, Cadieu CF, Solomon EA, Seibert D, DiCarlo JJ. Performance-optimized hierarchical models predict neural responses in higher visual cortex. Proc Natl Acad Sci. 2014;111(23):8619-24.

11. Deng J, Dong W, Socher R, Li L-J, Li K, Fei-Fei L. Imagenet: A large-scale hierarchical image database. In: 2009 IEEE conference on computer vision and pattern recognition. Ieee; 2009. p. 248-55.
