# Supplementary material for: Shrinking Bouma’s window: How to model crowding in dense displays
Source: PLoS Comput Biol. 2021 Jul 6;17(7):e1009187. doi: 10.1371/journal.pcbi.1009187 (PMC8284675; doi:10.1371/journal.pcbi.1009187)
Supplement: S5 Appendix — Detailed description of the model. (PDF) [file pcbi.1009187.s005.pdf]

S5 Appendix: Contour segmentation model ("Laminart")

The Laminart model (1) is a spiking neural network that computes illusory contours between aligned edges. In the model, grouping is crucial. Elements linked by any contour (illusory or real) are grouped together by dedicated neural populations. Stimuli are first segmented into different groups by the network's dynamics and, subsequently, elements within a group interfere (Fig A). Importantly, crowding is weak when the target belongs to a different group than most flankers, and strong otherwise. The segmentation process is triggered by local selection signals whose activity then spreads along connected contours. The location of the selection signals determines the output of the segmentation process.

It is very time consuming to run the model (for our displays, it would need to simulate several millions of spiking neurons for each display trial) and cannot go through the whole GA procedure in a realistic amount of time. However, exploiting the fact that the flankers are exclusively vertical or horizontal, we built a faster segmentation algorithm that reproduce the model behaviour for the displays used in Van der Burg et al. (2). For each display, the algorithm links neighbouring bars whenever they or their tips are aligned (Fig A, right). The different groups are defined as all disconnected sets of bars that are linked by the former procedure. This corresponds exactly to the behaviour of the full Laminart model but requires much less time to run.

At each trial, the algorithm sends selection signals that segment any group that is reached. In Francis et al. (1), because the visual stimuli tested with the model consisted of a vernier target flanked on both sides, two selection signals were sent at each trial, one on each side of the target. Here, because the flankers lie on all sides of the target, four selection signals are sent around the target at each trial. The segmentation layer that contains the target was used to

compute target-flanker interference. For each trial, the total interference, T, was defined exactly as in the Bouma model, and a choice was made about the orientation of the target, with a probability of correct response defined by Equation 1 in S1 Appendix. The only difference with the Bouma model is that, thanks to the segmentation process, a single gain A, was used for sparse and dense displays, without preventing the GA procedure to work. The performance for each display was defined as the fraction of correct responses over the trials.

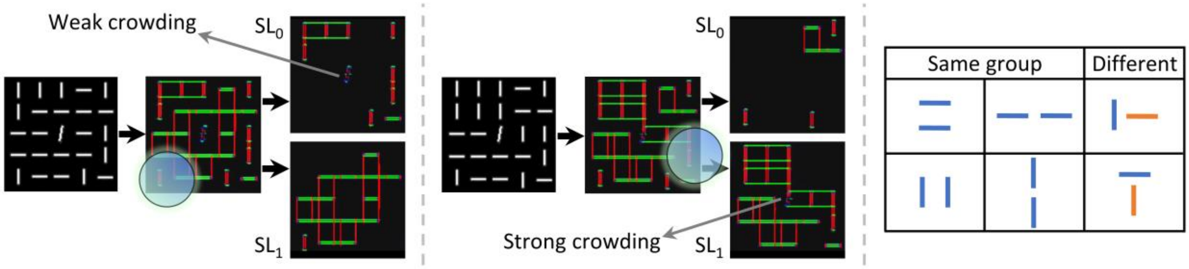

Fig A. Laminart model. In the original model (1), the stimulus is processed by an array of orientation-selective feature detectors. Coloured pixels that are depicted in the images correspond to the most active oriented cell at that location (red, green, blue, purple and turquoise for vertical, horizontal, oblique, almost vertical and almost horizontal orientations). Recurrent connections compute illusory contours between well-aligned edges. Elements that are linked by illusory contours belong to the same group. Then, local, top-down selection signals (blue circles) trigger a recurrent segmentation process, parsing the visual input in different segmentation layers (here, SL_0 and SL_1, but there can be more segmentation layers). After dynamic processing, all elements that are linked, through an actual or an illusory contour, to a location that is touched by a selection signal are parsed to the corresponding segmentation layer. Crowding is computed simply by applying the Bouma model to the segmentation layer that contains the target. **Left.** If only a few flankers are segmented with the target, crowding is weak. **Center.** If the target is linked with a large group of flankers through illusory contours, crowding is strong. **Right.** Because it would have taken too long to simulate the model for large displays, the segmentation process was replaced by an algorithm that reproduces its behaviour, given the simplicity of the stimuli involved in Van der Burg et al. (2). The algorithm assigns all elements to groups by linking pairs of well-aligned edges. After sending a selection signal, all groups of elements that are reached appear in the corresponding segmentation layer. The input of the grouping and segmentation algorithm is an array of 15 by 19 bits that encodes each flanker orientation, but the initial

selection signal process is simulated using an actual image of the stimulus. Spatial units in the model are defined by the size of the selection signals, which have a radius of 1 degree in the original version of the Laminart model. The resolution of the image, set to 15 pixels per degrees, is not crucial to the model but is used to determine whether or not a selection signal that partially overlaps with a flanker location would hit the flanker.

Results obtained with the Laminart model are shown in Fig 3 in the main text (6th row). The model reproduced Bouma's law simply because target-flanker interference was defined as in the Bouma model. The model reproduced human results for the proportion measure. During the GA procedure, performance increased with the generations. The selection measure revealed that the flanker locations that were crucial for this improvement were the target's nearest neighbours. This can be explained by the fact that, whenever all these crucial locations contain horizontal flankers, a "grouping shield" is created around the target (such as in Fig Aa, left), so that: a) no illusory contour can ever group flankers with the target; b) a segmentation signal has a large probability to hit a flanker that is linked to this shield, parsing many flankers to a different segmentation layer than the one of the target. For these reasons, the target's nearest neighbours were more crucial to determine crowding strength than in other models. In summary, this model replicated all human results well, but interference in the model was directly fitted to the sparse display data instead of proposing a mechanism.

References

1. Francis G, Manassi M, Herzog MH. Neural dynamics of grouping and segmentation explain properties of visual crowding. Psychol Rev. 2017;124(4):483.

2. Van der Burg E, Olivers CN, Cass J. Evolving the keys to visual crowding. J Exp Psychol Hum Percept Perform. 2017;43(4):690.
