# Supplementary material for: Shrinking Bouma’s window: How to model crowding in dense displays
Source: PLoS Comput Biol. 2021 Jul 6;17(7):e1009187. doi: 10.1371/journal.pcbi.1009187 (PMC8284675; doi:10.1371/journal.pcbi.1009187)
Supplement: S6 Appendix — Description of the model, including simulation of control models. (PDF) [file pcbi.1009187.s006.pdf]

S6 Appendix: Capsule network

Capsule networks are deep neural networks in which layers of neurons communicate through a recurrent process that implements grouping (Fig A). Each layer is made of many capsules, groups of neurons encoding specific features within their pattern of activity. Layers communicate through a time-consuming recurrent process called "routing by agreement" (1), in which each capsule in the lower layer predicts the activity of each capsule in the next layer. Grouping happens when many capsules agree that a certain higher-level capsule should be highly active: the corresponding higher-level capsule is activated and other higher-level capsules for which there is no agreement are shut down (Fig A, right). The entire network is trained end to end through backpropagation. Doerig et al. (2) showed that Capsule networks can explain uncrowding based on their grouping capabilities.

We trained the model for the GA procedure using a similar approach as in Doerig et al. (2). The Capsule network was first trained to recognize targets and groups of horizontal or vertical elements using a training set consisting of images that either contained a target in isolation or a rectangular array of 1 to 49 uniformly horizontal or vertical flankers. During the training phase, the Capsule network was also trained to discriminate between left and right targets (Fig A, left). The model was trained until it was able to classify the target with 67% of accuracy on a validation set composed of dense display arrays with 30% of vertical flankers. Note that only one of the 10 models we trained reached this performance level. After the training phase, this model was tested with sparse and dense displays. The performance was defined as the fraction of correct classifications over the trials. Note that only Bouma-sized crops were sent to the Capsule network during training, validation and testing. This was done for a better convergence

of the training loss and because the training process would have required too much memory to fit on our computer with full stimulus arrays.

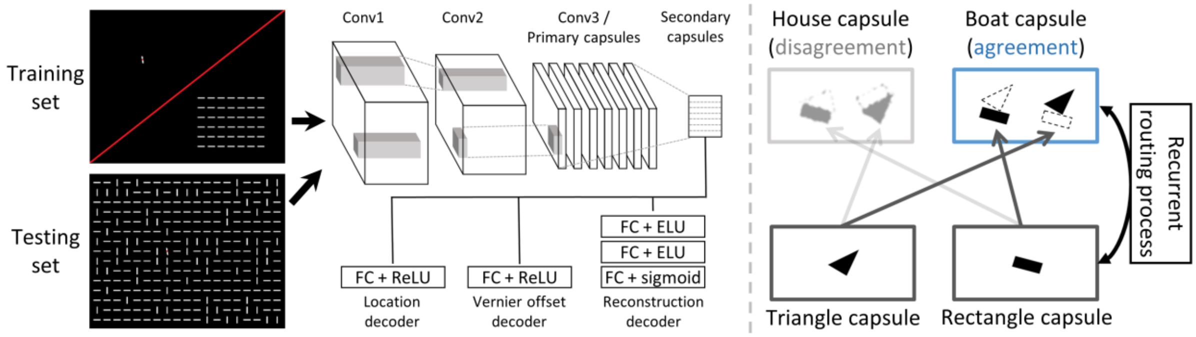

Fig A. Left. Capsule network. The input to the model is an actual image of the visual stimulus. Spatial units in the model are defined by the resolution of the stimulus, which was set to 15 pixels per degree (for computational reasons). The stimulus display is processed by a set of convolutional layers, conveying information to the primary capsules, which then projects to the secondary capsules. Routing by agreement happens between the primary and secondary capsules. In this case primary capsules encode visual elements (target, horizontal, vertical element) and the secondary capsules encode groups of visual elements. The output of the secondary capsules is sent to 3 different simple decoders (for stimulus reconstruction, stimulus location decoding and target orientation discrimination). The training set is composed of samples containing either the target alone or an array of exclusively vertical or horizontal flankers. The loss of the classifier is a combination of a reconstruction loss, and of cross-entropies on target classification and on stimulus location. In addition, a margin loss makes sure that the activity in the secondary capsules corresponds to the correct types of visual elements (target, horizontal, vertical group). After training the whole network end to end, we tested it with the four measures described in the Methods section, using the target orientation decoder to generate responses for each stimulus. **Right.** Routing by agreement. In this example, capsules in the lower layer encode basic shapes, and capsules in the higher layers encode objects. The activity pattern of each capsule encodes the characteristics of the input it is responsible for (size, location, orientation, etc.). Both primary capsule's outputs try to predict how activity is going to look in the secondary capsules. Because their predictions match in the boat capsule (dashed shapes vs. full shapes), the projection that lead to this agreement (dark arrows) is strengthened over time by the recurrent routing process. Because these same primary capsules do not agree with each other in the house capsule, this projection (light arrows) is weakened by the routing process. Adapted with permission from (2).

Results obtained with the Capsule network are shown in Fig 3 in the main text (7th row). Surprisingly, the model reproduced Bouma's law qualitatively simply by being trained at identifying targets and flankers (albeit unflanked performance is higher than in humans). The model reproduced human results for the proportion measure as well. The GA procedure improved the performance of the Capsule network along the generations, and the selection measure showed that the flanker locations that were crucial for this improvement were just above and below the target. In summary, this model replicated all human results well, except that only the flankers directly above and below the target (and not those to the left and right) are highlighted by the selection measure. One caveat is that only one out of the 10 models we trained reached good target discrimination in dense displays.

To control for the importance of segmentation processes in Capsule networks, we added simulations of control versions of the model (see Fig. B), as what was done in Doerig et al. (2). Importantly, these control versions contain the same number of parameters as in the capsule network but do not instantiate any grouping process. The first control version is a Capsule network in which the capsule layers are replaced by a fully connected feedforward layer, yielding a standard feedforward CNN with three convolutional layers and a fully connected layer. The second version is the same network as in the first control version, but with added lateral recurrent connections in the fully connected layer of the feedforward CNN, yielding a network with three convolutional layers followed by a fully connected recurrent layer. The third version is the same network as in the first control version, but with added top-down recurrent connections feeding back from the final fully connected layer of the feedforward CNN to the layer below, yielding a network with three convolutional layers followed by a fully connected layer that feed back into the previous one. The control models did not reproduce the human data, highlighting the importance of grouping processes in the Capsule network.

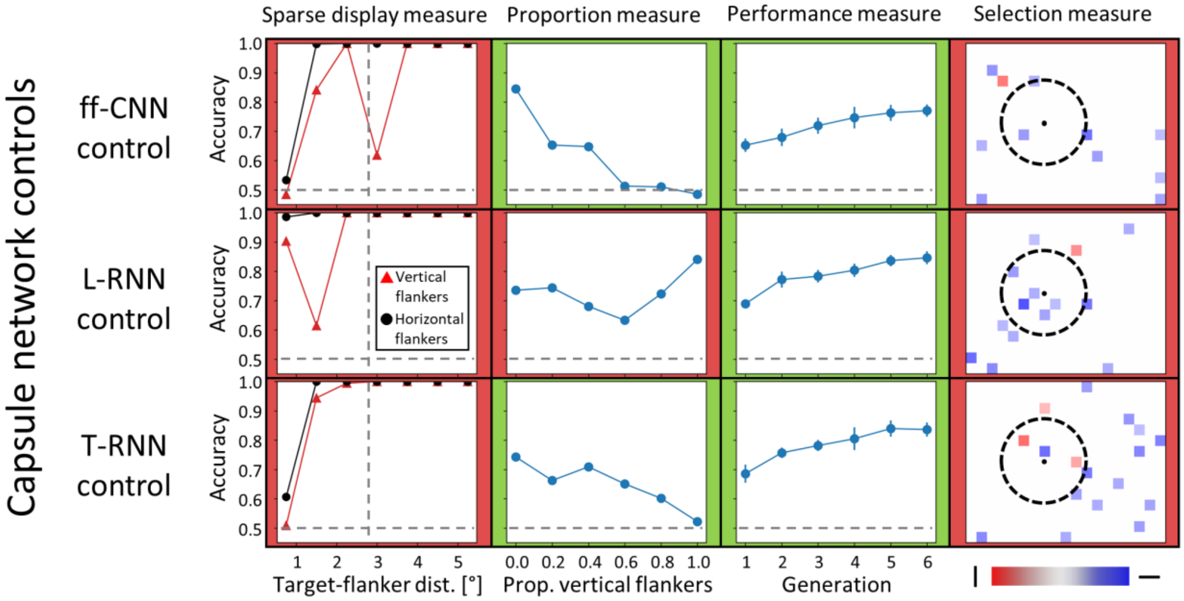

Fig B. Top. Results obtained with the feedforward CNN control version of the Capsule network model. **Center.** Results obtained with the lateral RNN control version of the Capsule network model. **Bottom.** Results obtained with the top-down RNN control version of the Capsule network model. The performance of all control models increases with the generations. However, all control models fail to reproduce the selection measure, as the GA procedure does not highlight any particular flanker location responsible for the performance improvements.

References

1. Sabour S, Frosst N, Hinton GE. Dynamic routing between capsules. In: Advances in neural information processing systems. 2017. p. 3856-66.

2. Doerig A, Schmittwilken L, Sayim B, Manassi M, Herzog MH. Capsule networks as recurrent models of grouping and segmentation. PLOS Comput Biol. 2020;16(7):e1008017.
