# Supplementary material for: Shrinking Bouma’s window: How to model crowding in dense displays
Source: PLoS Comput Biol. 2021 Jul 6;17(7):e1009187. doi: 10.1371/journal.pcbi.1009187 (PMC8284675; doi:10.1371/journal.pcbi.1009187)
Supplement: S7 Appendix — Detailed description of the model. (PDF) [file pcbi.1009187.s007.pdf]

S7 Appendix: Two-stage model ("Popart")

The contour segmentation model (Laminart model; 1) explained the configuration effects in dense displays very well, but the way to measure target-flanker interaction was simply to fit the experimental data of Van der Burg et al. (2) for sparse displays. On the other hand, the population coding model (3) naturally accounts for Bouma's law in sparse displays but does not replicate the preference measure in dense displays. For these reasons, we combined both models into a two-stage model (Fig A).

In this combination, the segmentation model acts as a grouping stage and selects *which* elements in the visual field are going to interfere with each other. Only the flankers that were parsed in the same group as the target are sent to the interference stage. The population coding model acts as an interference stage and determines *how* the elements that were selected during the first grouping stage interfere. The parameters of both models were kept the same as in their respective descriptions above. The only difference was that the performance measure of the segmentation model was now computed by feeding the content of the target's segmentation layer to the population coding model.

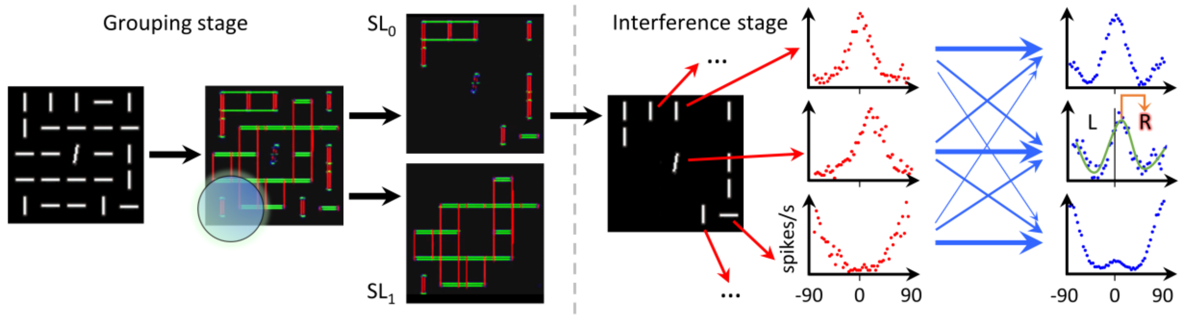

Fig A. Popart model. The model is composed of two stages. **Left.** Grouping stage. The Laminart model algorithm is used to parse the stimulus in different segmentation layers. **Right.** Interference stage. From the output of the

segmentation algorithm, a new stimulus is built. Only the elements present in the segmentation layer that contains the target are processed by the population coding model to generate a response.

Results obtained with the model are shown in Fig 3 in the main text (last row). Thanks to the combination of both segmentation and population coding models, the Popart model qualitatively reproduces human results for all measures.

References

1. Francis G, Manassi M, Herzog MH. Neural dynamics of grouping and segmentation explain properties of visual crowding. Psychol Rev. 2017;124(4):483.

2. Van der Burg E, Olivers CN, Cass J. Evolving the keys to visual crowding. J Exp Psychol Hum Percept Perform. 2017;43(4):690.

3. Van den Berg R, Roerdink JB, Cornelissen FW. A neurophysiologically plausible population code model for feature integration explains visual crowding. PLoS Comput Biol. 2010;6(1):e1000646.
