# Supplementary material for: Shrinking Bouma’s window: How to model crowding in dense displays
Source: PLoS Comput Biol. 2021 Jul 6;17(7):e1009187. doi: 10.1371/journal.pcbi.1009187 (PMC8284675; doi:10.1371/journal.pcbi.1009187)
Supplement: S8 Appendix — Detailed description of the experiment. (PDF) [file pcbi.1009187.s008.pdf]

S8 Appendix: Human experiment for proportion measure

We asked human participants to sit at 62 cm from an LCD screen (120 Hz refresh-rate), in a dimly lit room. The experiment was programmed and run using OpenSesame (1). The task was to discriminate between a left or a right target (tilted by 5°) presented in the periphery of the visual field. At each trial, as was done in Van der Burg et al. (2), the location of the target was either on the left or on the right of a central white fixation dot (0.1° radius). The possible target locations were indicated by two red dots (0.02° radius). These dots were visible during the whole experiment. When displayed, the target was embedded in an array of 15 rows by 19 columns of vertical or horizontal flankers (dense display, see Fig 2b in the main text). The target was always displayed at 6° of eccentricity (8th row, 8th column in the flanker array). A trial consisted of 500 ms during which the white and the red dots were presented alone, followed by 150 ms in which the target and the flanker array appeared, followed by an unlimited amount of time in which the observers could give their response by pressing a key. After the response was recorded, a new trial was initiated. The experiment consisted of 11 blocks (1 for practice) of 24 trials each. In each block, trials for each condition (0%, 20%, 40%, 60%, 80% or 100% of vertical elements in the flanker array) were mixed and evenly distributed (i.e., 6 trials per condition). At the end of each block, feedback was given to the observer as the proportion of correct responses in the performed block. We ran 7 participants in total, but we discarded 1 participant who was at chance level for all conditions. Results are shown in Fig 3 in the main text (top row, 2nd column). Participants gave oral consent before the experiment, which was conducted in accordance with the Declaration of Helsinki except for the preregistration (World Medical Organization, 2013) and was approved by the local ethics committee (Commission

d'éthique du Canton de Vaud, protocol number: 164/14, title: Aspects fondamentaux de la reconnaissance des objets protocole général).

References

1. Mathôt S, Schreij D, Theeuwes J. OpenSesame: An open-source, graphical experiment builder for the social sciences. Behavior research methods. 2012;44(2):314-24.

2. Van der Burg E, Olivers CN, Cass J. Evolving the keys to visual crowding. Journal of Experimental Psychology: Human Perception and Performance. 2017;43(4):690.
