# Supplementary material for: Shrinking Bouma’s window: How to model crowding in dense displays
Source: PLoS Comput Biol. 2021 Jul 6;17(7):e1009187. doi: 10.1371/journal.pcbi.1009187 (PMC8284675; doi:10.1371/journal.pcbi.1009187)
Supplement: S9 Appendix — (PDF) [file pcbi.1009187.s009.pdf]

S9 Appendix: Pooling model controls

In Fig 3 (main text), we tuned the model parameters to reproduce Bouma's law in sparse displays and showed that only the grouping models can shrink Bouma's window to the nearest neighbour distance in dense displays. Here, we included control simulations in which we instead tuned the parameters of the pooling models to reproduce human behaviour in dense displays (selection measure) and then examined their behaviour using sparse displays (Fig A). Each model's pooling range was decreased to the nearest neighbour distance by tuning the relevant parameters. For the Bouma model and the Popcode model (see S1 and S2 Appendices for more details), the pooling range is simply a scalar parameter that we modified. For the texture model (see S3 Appendix for more details) in which Bouma-sized crops of the stimuli were used, we simply used smaller crops. For the CNN classifier (see S4 Appendix for more details), we trained a classifier to decode left and right targets from the activity of the second layer of the CNN, whose receptive field size matches the nearest neighbour distance.

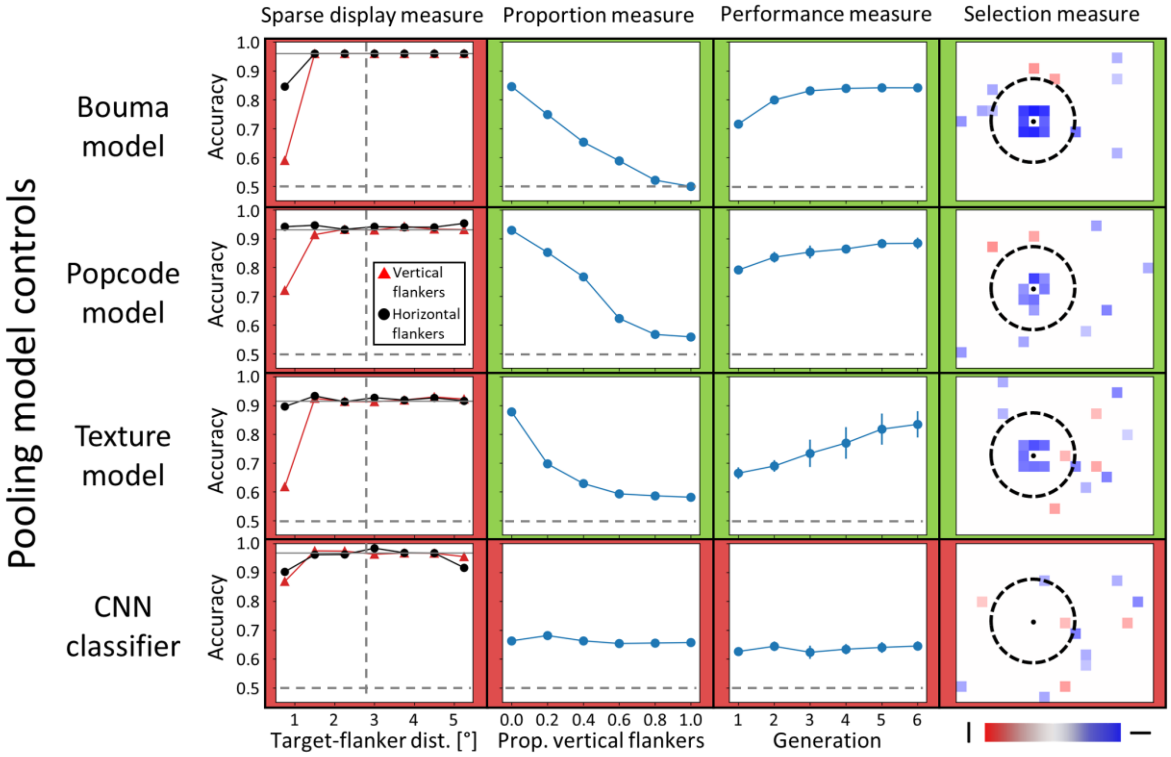

Fig A. Results obtained with the control simulations of the pooling models, in which the pooling range was adapted to reproduce human behaviour in the selection measure instead of the sparse display measure. The description of each measure is the same as in Fig 3 (main text). Except for the CNN classifier, in which the GA procedure did not improve performance, it was possible to adapt the interference range of the pooling models to reproduce human behaviour in the selection measure (the GA procedure improved performance, highlighting mostly the target nearest neighbours). However, the interference range was also shrunk in the sparse display measure (in sparse displays, performance was impaired only when the flankers were very close to the target).

The results in Fig A show that tuning pooling models to shrink Bouma's window in dense displays prevents them to reproduce Bouma's law in sparse displays. Indeed, the range of interference in sparse displays is also shrunk to the nearest neighbour distance, in contrast to the models that include a grouping component (see Fig 3). These results provide further evidence that a grouping stage is necessary to exhibit a small range of interference in dense display, while keeping a Bouma-sized range in sparse displays.
