# Supplementary material for: Shrinking Bouma’s window: How to model crowding in dense displays
Source: PLoS Comput Biol. 2021 Jul 6;17(7):e1009187. doi: 10.1371/journal.pcbi.1009187 (PMC8284675; doi:10.1371/journal.pcbi.1009187)
Supplement: S10 Appendix — Assessment of the similarity between the model results and the human data, for Fig 3. (PDF) [file pcbi.1009187.s010.pdf]

S10 Appendix: Quantitative similarity measurements

In Fig 3 (see main text), we compared how the different models we tested reproduce human behaviour in sparse and dense displays. To compare the different models, we qualitatively assessed the similarity between each model's measures and the corresponding human data. In Fig A, for completeness, we include *quantitative* assessments of the similarity between model and human behaviour. The similarity between the sparse display, proportion and performance measures and the corresponding human data was computed as the Pearson correlation coefficient between model and human performance. For the selection measure, we used the structural similarity index (1) because the output of this measure is a 2-dimensional image.

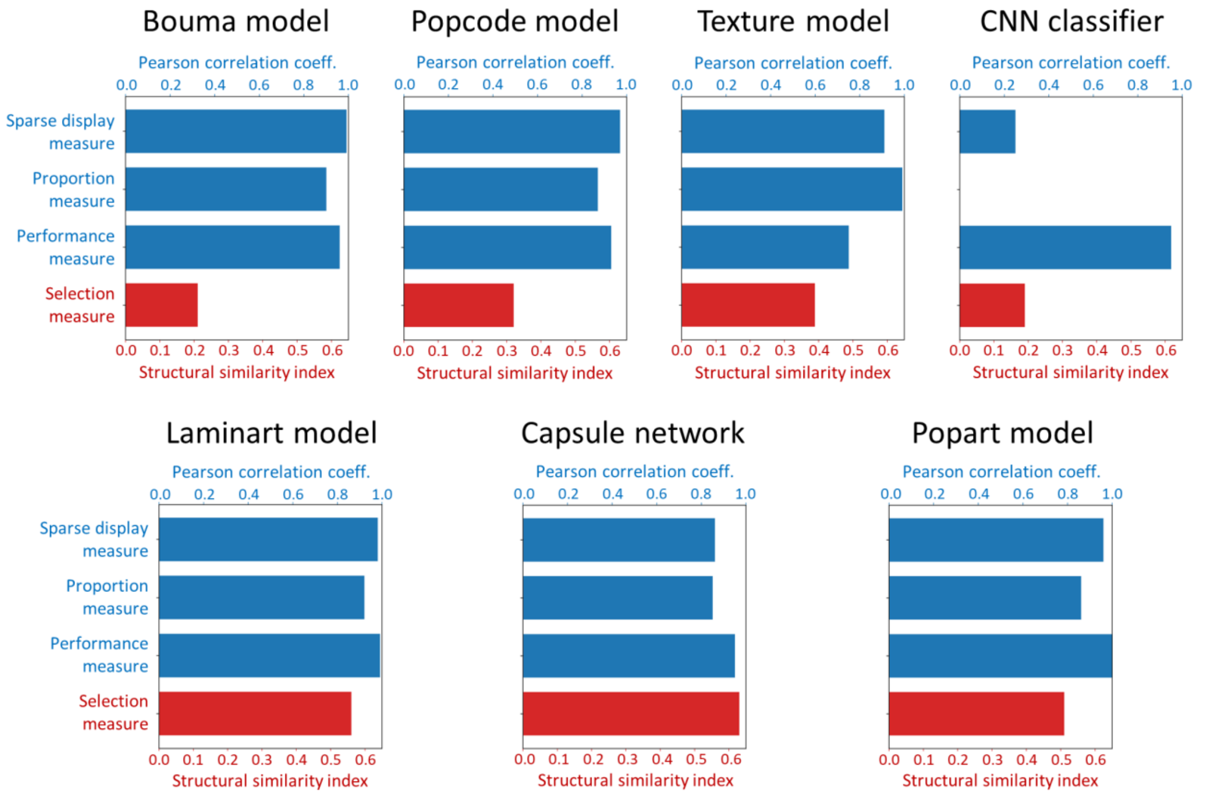

Fig A. Quantitative assessment of the similarity between the model results and the human data, for the different measures presented in Fig 3 (see main text). As described qualitatively in Fig 3, all models (except for the CNN classifier) reproduce human behaviour for the sparse display, proportion, and performance measures, but only

the grouping models are able to reproduce human behaviour in the selection measure as well. Here, this claim is confirmed by our quantitative assessment method. Overall, the models obtain high and comparable similarly scores for the sparse display, proportion, and performance measures (except for the CNN classifier). However, the grouping models overall obtain higher similarity scores than pooling models for the selection measure.

References

1. Wang Z, Bovik AC, Sheikh HR, Simoncelli EP. Image quality assessment: from error visibility to structural similarity. IEEE Trans Image Process. 2004;13(4):600-12.
