# Supplementary material for: Shrinking Bouma’s window: How to model crowding in dense displays
Source: PLoS Comput Biol. 2021 Jul 6;17(7):e1009187. doi: 10.1371/journal.pcbi.1009187 (PMC8284675; doi:10.1371/journal.pcbi.1009187)
Supplement: S11 Appendix — Detailed version of the rightmost column of Fig 3. (PDF) [file pcbi.1009187.s011.pdf]

S11 Appendix: Fine-grained version of the selection measures

In Fig 3 (see main text), the results of the selection measures are presented by cutting the values for which neither vertical nor horizontal flankers were significantly overrepresented by the GA procedure, compared to a random selection process. This allowed to highlight the range of interaction between the target and the flankers in dense displays. For completeness, we include a "fine-grained" version of these measures (for the human data as well as for the model results), in which the values are not put down to zero if not statistically significant (Fig A).

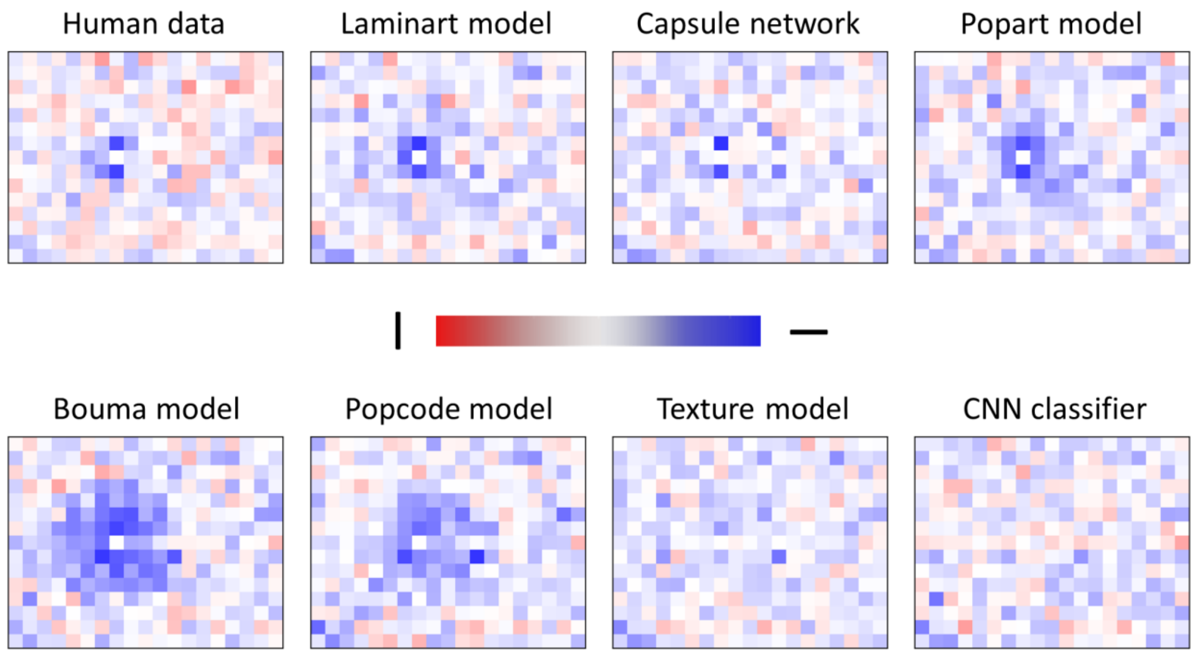

Fig A. Fine-grained version of the selection measures presented in Fig 3 (see main text). A red or a blue slot respectively indicate, for each location of the dense display, the fraction of vertical or horizontal flankers that were selected by the GA procedure, compared to randomly selected displays, after 6 generations.
