# Supplementary material for: Shrinking Bouma’s window: How to model crowding in dense displays
Source: PLoS Comput Biol. 2021 Jul 6;17(7):e1009187. doi: 10.1371/journal.pcbi.1009187 (PMC8284675; doi:10.1371/journal.pcbi.1009187)
Supplement: Attachment — Submitted filename: Response_To_Reviewers.docx [file pcbi.1009187.s012.docx]

We thank the editor and both reviewers for their feedback, which helped us improve and clarify the manuscript. We answered to all the reviewers’ comments by substantially reworking the discussion and conducting new simulation experiments. In particular, we conducted 3 controls using CNNs and recurrent CNNs for the capsule network, as well as control simulations for each of the 4 pooling models. We also reran a subset of our simulations with more subjects, as proposed by reviewer 1. Note that in the manuscript, we changed the name of the preference measure to selection measure, for clarity. In addition, we carried out some minor improvements to the figure layouts and the language. These improvements do not alter the points we make, but they improve clarity. Also note that we were asked to create a different version of Fig 1a for copyright reasons.

In the following, we respond to each comment of the reviewers in detail. The reviewers’ comments are in **bold** and our response are in normal font. When necessary, we copied in *italic* the text that we added to the revised manuscript.

**Reviewer #1**

**Bornet et al. investigate how well different models of crowding reproduce the pattern of results from an experiment with human observers. The human observer data stems from an ingenious published experiment, which instead of using simple sparse displays, uses a dense grid of stimuli which is modified over several generations of a genetic algorithm to minimise crowding. In the present manuscript, the authors let the responses of the different models drive the input to the genetic algorithm instead. The key finding is that all four ‘pooling’ models fail to reproduce aspects of the human data, while all three models which include a grouping mechanism produce much closer correspondence.**

**I read this manuscript with great interest. It is clearly written throughout and the conclusions are well-supported by the results of the simulations. I have no serious concerns. I do however have a few suggestions which might improve the manuscript further:**

**1) Some of the models show substantial variation in the measures depicted in Fig.3. As only 10 ‘participants’ were simulated for each, the results for some of the models are likely to vary quite a bit if the simulation is rerun. As far as I understand, limiting sample size to the human data is necessary to reproduce the ’preference measure’ (rightmost column) as this is based on statistical testing and thus depends on sample size. This is however not the case for the other columns. Would it be possible to base the three leftmost columns on a higher number of simulations to provide clearer data?**

Thank you for this very good idea. We re-ran the sparse display and proportion measures (first and second columns in Fig 3) with more model “participants” to avoid variability if the measures were repeated. We included the updated simulation results for the sparse displays and proportion measures in Fig 3 and adapted the text accordingly. The performance measure (3rd column in Fig 3), however, is also based on statistical testing: it depicts the performance of the model at each generation in the GA procedure, producing the results in the preference measure (now called selection measure; 4th column in Fig 3). Note that the more precise results that we obtained in the sparse display measures allowed us to finetune the parameters of the models for a better fit of the human data (Bouma’s law). We hence re-ran the GA measures for all models (but with 10 model subjects). Overall results did not change.

**Perhaps the preference measures derived from those simulations could in addition also be depicted without ‘cutting’ by means of statistical tests to provide more fine-grained information on the range of interference in each model.**

Thank you for this proposition. We think that cutting the values for which vertical/horizontal flankers are not significantly preferred better features the behaviour of the models, since it highlights the range of interaction between the target and the flankers, which is a crucial property of visual crowding. Nevertheless, we added the fine-grained version of these measures as an appendix (S11 Appendix) and referred to it the caption of Fig 3.

**2) The different models do not only vary in how well they reproduce the means of the human data, but also in how well they reproduce the standard deviations of the human data (e.g. most of them produce much higher variability in the proportion measure than human observers). The interpretation of the models is however only based on the means, not the standard deviations. I would like the authors to either justify why the models’ ability to reproduce the standard deviations is less important/informative/valid or alternatively, include it in the interpretation of their results.**

Thank you for noticing this. For the human measures, the variability comes both from the differences between participants and from internal noise. For the model measure, the variability comes only from external noise (e.g. select a right or left target with a certain probability that depends on sampling noise and not on the model parameters, once the probability is set). Because the sources of noise are different, we think that analysing how well models reproduce the standard deviations in the human data might not be very insightful. Hence, we would like to stick to your former idea (see your point number 1) and have more model “participants“ in the sparse display and proportion measures (very small standard deviations since only one set of parameters is selected for each model, which would not happen for human participants).

**3) Can the models be evaluated in terms of biological plausibility (the extent to which the computations are compatible with what is known about the visual cortex)? For example, is it plausible that the computationally more demanding models could be implemented in the cortex in such a way as to be compatible with human behavior when stimuli are only presented briefly? Some consideration of this may be worth including in the discussion.**

Thank you for this idea. Of course, biological plausibility is very ill-defined. For example, on the one hand, feedforward CNNs could be ruled-out from the beginning because the human visual cortex is highly recurrent. On the other hand, they explain neural activities well, once trained on image recognition. As another example, depending on how biological plausibility is defined, the Popart model may not be the best since segmentation and interference between visual elements happen at two separate stages, which might not be the case in the human brain. On the other hand, population coding is a well characterized feature of human visual processing, and so is visual segmentation. For these reasons, we do not think that enough is known about biological circuits to arbitrate between the different models we use.

**4) The presented simulations yield an argument that grouping may be a sufficient condition for capturing important aspects of human-like behavior in models of crowding. But is it necessary? Put differently, out of the infinity of possible crowding models, are there plausible alternatives to grouping that also yield e.g. a shielding of the target from interference from further away flankers in some conditions (as described in the supplementary material)?**

Thank you for this comment. Our main idea is that models should not only account for the shrinking of Bouma’s window in this particular paradigm but also for other characteristics of crowding, such as Bouma’s law in sparse displays. Of course, we could come up with feedforward networks that only consider the closest neighbours and this model would explain the dense display results, but they would not explain any of the former characteristics. For this reason, we chose to use models that are well-known to explain characteristics of crowding and/or properties of vision in general and tested how they generalize to crowding in dense displays. For example, CNNs reach human level performance on various complex visual tasks and are subject to crowding. Summary statistics models can explain how humans process complex images without undergoing cognitive overload and capture many characteristics of visual crowding. Segmentation processes are important to solve ill-posed problems of vision and capture the effects of flankers that lie beyond Bouma’s window in crowding (e.g. uncrowding). The goal is not to overfit on a particular paradigm, but to test how processing characteristics of these different hypotheses generalize to this new paradigm (crowding in dense displays). Hence, our main message is not that grouping and segmentation processes are necessary to shrink Bouma’s window in dense displays, but that models including such processes are the best at explaining crowding overall. We clarified this in the discussion:

*Of course, many other models could potentially address these results. For example, we could train a feedforward neural network to only consider the nearest neighbour flankers, since feedforward networks are universal function approximators (49). However, such a model would be scientifically stale, because crowding is better seen as a probe into visual processing rather than as an explanatory goal per se. A model that only explains this paradigm is useless. For example, feedforward models tuned to explain the shrinking of Bouma’s window in the selection measure do not reproduce Bouma’s law in sparse displays (see S9 Appendix). The goal is not to overfit on a particular paradigm, but to test how processing characteristics of different hypotheses generalize to this new particular paradigm (crowding in dense displays). CNNs reach human level performance on various complex visual tasks and are subject to crowding. Summary statistics models can explain how humans process complex images without undergoing cognitive overload and capture many characteristics of visual crowding. Segmentation processes are important to solve ill-posed problems of vision and capture the effects of flankers that lie beyond Bouma’s window in crowding (e.g. uncrowding). Each of these modelling frameworks has been fruitful in other areas and the goal is to test how they generalize to crowding in dense displays, to uncover strengths and weaknesses of each approach.*

*Along the same lines, Manassi et al. (35) showed that elements beyond Bouma’s window can have a strong impact on target discrimination, and that the configuration of elements in the whole visual field determines crowding strength (see also 24,25). A similar extensive comparison of models showed, once again, that only models that could reproduce these results contained a dedicated grouping stage (46; see also 34,43,49). Moreover, Van der Burg et al. (51) showed that crowding in dense displays does not depend on target eccentricity but only on the configuration of the nearest neighbours. The grouping models that we tested here can exhibit uncrowding at the same time as the shrinking of Bouma’s window, depending on the specific configuration of the flankers. In contrast, a model that modulates the window of integration based on the number of flankers but not their configuration, such as divisive normalization (52), will not be able to explain why faraway elements interact only in certain cases (e.g. why Manassi and colleagues found very long ranging interactions but Van der Burg and colleagues found the opposite). For all these reasons, it becomes clear that grouping, and not Bouma’s window, determines which elements interfere with each other in human vision. In summary, our results do not prove that grouping and segmentation processes are the only way to shrink Bouma’s window in dense displays, but rather show that they are the best at explaining crowding overall.*

**- Lines 187-190: The descriptions here belong in a figure legend rather than the main text**

Thank you. We changed the text accordingly.

**- Lines 203-204: ‘If any model was far from this 67% requirement’: How far is ‘far’?**

We agree that this statement needs clarification. Our method was first to tune the model parameters so that the performance was as close as possible to 67% in dense displays. However, in some cases, model performance could not reach this level at all, i.e. it was lower than 67% (a necessary condition for the tuning is that the model is able to go above and below 67%, following the parameter choice). Only in these cases, we modified the target orientation amplitude to help the model reaching the required performance for an optimal GA procedure. We added more details about this in the Methods section:

*The performance of some models was bounded by a value lower than 67%. Only in these cases, we adapted the target orientation amplitude so that higher performances than 67% could be reached, thereby allowing the model parameters to be tuned to the required level of performance.*

**Reviewer #2**

**I found this article interesting, in that it uses a display that differs from the one that has repeatedly been used by some of the authors for making similar points. The main message of the article, i.e. the necessity for a grouping process that is not present within typical feedforward architectures, has already been made by some of the authors in previous publications (e.g. Doerig et al 2020 in this same journal), however using uncrowding displays. In this study, the same result is demonstrated using a different class of displays, namely dense arrays of oriented segments that could take on several configurations.**

**My main issue with this article is that I am not convinced that such a simple set of psychophysical results cannot be explained by a relatively simple model. Unfortunately I don't have time to try and model it myself with code, but the nature of the human results makes me suspect that there should be a way to model them without the necessity for complex algorithms like capsule networks. This is in contrast to uncrowding, a phenomenon for which a relatively low-level strategy seems bound to fail. In other words, while I am more open to the idea that uncrowding may require relatively sophisticated strategies and the grouping idea makes sense, I find it more difficult to convince myself that this is the case for the human data presented here.**

Thank you for raising this concern, which helped us clarify our arguments. Our main idea is that models should not only account for the shrinking of Bouma’s window in this particular paradigm but also for other characteristics of crowding and vision in general. Of course, we could come up with feedforward networks that only consider the closest neighbours and this model would explain the dense display results, but they may fail to explain other hallmarks of crowding, such as Bouma’s window in sparse displays or uncrowding effects. In fact, we now show (see S9 Appendix) that it is of course possible to tune the parameters of all the pooling models to reproduce the shrinking of Bouma’s window in dense displays (preference measure; now selection measure), but doing so prevents them from reproducing human behaviour in sparse displays (Bouma’s law), since only nearby elements can ever interact. As far as we know, only the grouping models can reproduce both measures, as depicted in Fig 3.

Here, we chose to test models that are well-known to explain certain features of crowding and/or properties of vision in general and tested how they generalize to crowding in dense displays. For example, CNNs reach human level performance on various complex visual tasks and are subject to crowding. Summary statistics models can explain how humans process complex images without undergoing cognitive overload and capture many characteristics of visual crowding. Segmentation processes are important to solve ill-posed problems of vision and capture the effects of flankers that lie beyond Bouma’s window in crowding (e.g. uncrowding). The goal of our study is not to overfit on a particular paradigm, but to test how the processing characteristics of these different models generalize to crowding in dense displays. Here, we do not claim that grouping and segmentation processes are the only way to shrink Bouma’s window in dense displays. We rather show that they are the best at explaining crowding overall. We clarified these points in the discussion:

*Of course, many other models could potentially address these results. For example, we could train a feedforward neural network to only consider the nearest neighbour flankers, since feedforward networks are universal function approximators (49). However, such a model would be scientifically stale, because crowding is better seen as a probe into visual processing rather than as an explanatory goal per se. A model that only explains this paradigm is useless. For example, feedforward models tuned to explain the shrinking of Bouma’s window in the selection measure do not reproduce Bouma’s law in sparse displays (see S9 Appendix). The goal is not to overfit on a particular paradigm, but to test how processing characteristics of different hypotheses generalize to this new particular paradigm (crowding in dense displays). CNNs reach human level performance on various complex visual tasks and are subject to crowding. Summary statistics models can explain how humans process complex images without undergoing cognitive overload and capture many characteristics of visual crowding. Segmentation processes are important to solve ill-posed problems of vision and capture the effects of flankers that lie beyond Bouma’s window in crowding (e.g. uncrowding). Each of these modelling frameworks has been fruitful in other areas and the goal is to test how they generalize to crowding in dense displays, to uncover strengths and weaknesses of each approach.*

*Along the same lines, Manassi et al. (35) showed that elements beyond Bouma’s window can have a strong impact on target discrimination, and that the configuration of elements in the whole visual field determines crowding strength (see also 24,25). A similar extensive comparison of models showed, once again, that only models that could reproduce these results contained a dedicated grouping stage (46; see also 34,43,49). Moreover, Van der Burg et al. (51) showed that crowding in dense displays does not depend on target eccentricity but only on the configuration of the nearest neighbours. The grouping models that we tested here can exhibit uncrowding at the same time as the shrinking of Bouma’s window, depending on the specific configuration of the flankers.*

**As I said, I have no time to model it myself, but I would like to propose some possibilities. For example, suppose we entertain the notion of a flexible attentional window. One model that may provide a bottom-up procedure for shrinking/enlarging the window is the normalization model by Reynolds and Heeger. It may well be that this model makes predictions that are inconsistent with the human data presented here, but perhaps variants of normalization may work. What I have in mind is a situation where the attentional window is relatively large in a sparse display, and then by adding stimulus items, these items stimulate a suppressive attentional field that narrows the focus of attention around the target bar.**

Thank you for proposing this model. First, we now explicitly mentioned in the manuscript that we share the code to test any model with the genetic algorithm procedure. In addition, in Doerig et al. (2019), we also shared the code to generate uncrowding stimuli, so that any model successfully capturing human behaviour in dense displays can be tested on uncrowding stimuli. To explain the previous and the present results showing that Bouma’s window is a myth, we agree that other models are possible as well, e.g., using attention to modulate the size of the interference window. Nevertheless, the conclusion remains that the global configuration must have an effect on which elements interfere.

For the bottom-up part of your comment, we would like to argue that if a model can shrink Bouma’s window using a bottom-up global normalization process, then it cannot explain the beneficial influence of faraway elements in other cases, such as in uncrowding (see Doerig et al., 2019, 2020). Our point is that the grouping models that we tested here can exhibit uncrowding at the same time as the shrinking of Bouma’s window, depending on the specific configuration of the flankers. In other words, these models can account for a flexible pooling range, i.e., depending on the specific configuration of the flankers. This would not be the case with a global normalization process that would depend on the mere amount of flankers and not on subtle details of the flanker configuration. We thank you very much for this comment as it helped us clarify our point. We added a paragraph in the discussion about your idea of using a global normalization process to shrink the interaction window in dense displays:

*In contrast, a model that modulates the window of integration based on the number of flankers but not their configuration, such as divisive normalization (52), will not be able to explain why faraway elements interact only in certain cases (e.g. why Manassi and colleagues found very long ranging interactions but Van der Burg and colleagues found the opposite). For all these reasons, it becomes clear that grouping, and not Bouma’s window, determines which elements interfere with each other in human vision. In summary, our results do not prove that grouping and segmentation processes are the only way to shrink Bouma’s window in dense displays, but rather show that they are the best at explaining crowding overall.*

**Another possibility is the following: suppose my attentional field is driven by a simple heuristic, like "find the centre of the display: that's where the target is". For a sparse display, my estimate of the centre will be very uncertain/inaccurate, so it will be necessary to maintain a relatively large uncertainty/attentional window. For a dense display, where the stimulus items basically trace out the square for me, estimating the centre can be done easily and with high accuracy. A process of this kind can be implemented using low-level algorithms.**

Thank you for mentioning this possibility. However, we do not believe this would work. First, the target is not in the centre of the flanker array. It is at the 8th row and 8th column of a 15 rows by 19 columns flanker array. A large display cueing the 8/19th of a grid sounds unrealistic. In fact, we might argue the opposite: in the sparse displays that are used in this manuscript (and in the original data), the left and right flankers precisely indicate the horizontal position of the target, and the top and bottom flankers precisely indicate its vertical position. Unless we have a well-defined model of spatial cueing, arguments of this kind are too handwavy to be tested directly; but we added a passage mentioning this possibility in the discussion:

*Importantly, the shrinking of Bouma’s window in dense displays cannot be explained by the large flanker array providing a spatial cue towards the target’s location. Indeed, the target is not in the centre of the flanker array. It is at the 8th row and 8th column of a 15 rows by 19 columns flanker array.*

We would like to add that we just submitted a paper addressing this kind of cueing argument thoroughly, and why we think it fails (but this is beyond the scope of the present paper).

**There are other possibilities. My point is that I am worried that the authors may not have exhausted all reasonable explanations based on a simpler model. I understand that it is difficult to exclude all such models, as there is potentially an infinite number of them, but I would like to be convinced that one really needs a different class of models to explain this human pattern.**

Thank you, we hope that the above (and below) points satisfied your concerns.

**My comments above are also relevant to Figure 3. One suggestion I have with relation to this Figure is that the authors should include a simulation where the "pooling models", or at least some of them, are optimized to replicate the preference measure, so as to demonstrate that this will result in a failure to replicate the other measures. This is important for clarity: some readers may find it puzzling that a simple human pattern like the one in the last column cannot be reproduced, for example, by a CNN. The point is that it cannot be reproduced while at the same time reproducing the sparse-display measure, but I would try and make this message clearer with an explicit simulation, possibly in a separate figure: for example you could have a figure where you present only two simulations for the same pooling model, one simulation for which the model is tailored to the sparse measures, and another one where it is tailored to the dense measures. This figure should demonstrate that you can simulate one or the other, but not both.**

Thank you for raising this point. We added an appendix containing a figure in which we tune the parameters of the pooling models to reproduce the human data in the preference measure (now selection measure). We referred to it in the caption of Fig 3. We show that tuning the pooling models to shrink Bouma’s window in dense displays prevents them to fit Bouma’s law in sparse displays (see S9 Appendix).

**I also see issues with the CNN simulations. The authors adopt a slightly unconventional approach, in which they build a read-out module on top of each layer, train that module for a pre-trained Alexnet model, and then choose the layer for which the trained read-out module produces a good match with the sparse measure. I don't have a particular problem with this, but there are many other sensible choices that may produce substantially different results. For example, what happens if you choose the preference (dense) measure as your target and compute cost with respect to that? Will that produce a good match with the sparse measure for free? What if you do this by transfer-learning only the last layer via reducing the fc layer output, as commonly done?**

Thank you for this idea. However, in practice, we think it is not possible to use the preference measure (now selection measure) for the CNN loss. Indeed, the training process of the classifier would need to store the forward pass of the entire GA procedure in the VRAM of the GPU (times the batch size) before a backpropagation step is performed. Even assuming a batch size of 1, this seems way too large to fit on our devices (a single GA procedure consists of 38400 stimuli shown to the network). In addition, it is unclear whether the training process would converge (and, if yes, in how much time). Finally, even if the training process could theoretically converge, the time needed for this procedure would be unpractical. A single GA runs takes several minutes to run with the AlexNet classifier. Hence, assuming a lower bound of 100’000 GA runs performed by the model to converge to an optimum, the training process would last for months or years. Using transfer-learning is impractical too, since it would still require fitting neural activities of the CNN throughout the entire GA procedure on the GPU. Lastly, as explained above, and as we now mention in the discussion (paragraph copied above for one of your previous comments), our aim is not to overfit on this particular paradigm, but to uncover general principles of crowding and vision. This method would fail to do so.

**There are many other issues with the section on CNN. Only Alexnet is used. I agree that this is a good example of CNN, but some subsequent architectures cannot be viewed as mere derivatives of Alexnet, and one does not have to look far: even the classic resnet and VGG families have added new features.**

First, we agree that focusing only on AlexNet might not be a fair comparison against the other type of deep network that we ran in this study, i.e., the capsule network. For this reason, we added simulations for control versions of the capsule network in the manuscript, as what was done in Doerig et al. (2020). The first control version is a capsule network in which the capsule layers are replaced by a fully connected feedforward layer, yielding a standard feedforward CNN with three convolutional layers and a fully connected layer. The second version is the same network as in the first version, but with added lateral recurrent connections in the fully connected layer of the feedforward CNN, yielding a network with three convolutional layers followed by a fully connected recurrent layer. The third version is the same network as in the first control version, but with added top-down recurrent connections feeding back from the final fully connected layer of the feedforward CNN to the layer below, yielding a network with three convolutional layers followed by a fully connected layer that feed back into the previous layer. These control versions contain the same number of parameters as in the capsule network, but do not instantiate any grouping process. We added a figure to the appendix of the capsule network model (S6 Appendix), in which the control networks are tested as well.

Second, there are of course different architectures for feedforward CNNs, such as ResNet and VGG. However, we would like to argue that the problem with these networks is not the kind of feedforward CNN, but the fact that these networks are based on pooling, as thoroughly investigated in Doerig et al. (2020). In summary, neither AlexNet nor the capsule networks feedforward CNNs trained on target recognition could explain the shrinking of Bouma’s window, and we have strong theoretical reasons to believe that other ffCNNs would fail too. Moreover, Geirhos et al. (2020) showed that CNNs are remarkably consistent with one another when tested behaviourally, irrespective of architecture. In conclusion, although we cannot test all possible feedforward CNNs, we have good grounds for proposing that they cannot explain the flexible range of Bouma’s window. We added a paragraph in the discussion to explain this:

*There are many more architectures for feedforward CNNs, such as ResNet and VGG. We think that these networks face similar problems as the CNNs tested here because they are also based on pooling. Because of this pooling, performance always deteriorates when flankers are added, irrespective of the global configuration of elements (for an in-depth argument, see Doerig et al., 34). In support of this claim, neither AlexNet (see S4 Appendix) nor the capsule networks controls (see S6 Appendix, feedforward and recurrent CNNs) can explain the shrinking of Bouma’s window. In addition, Geirhos et al. (53) showed that CNNs are remarkably consistent with one another behaviourally, irrespective of architecture. In summary, we cannot test all possible models, but have good grounds for proposing that CNNs cannot explain the flexible range of Bouma’s window.*

**It would be reassuring to see more of an effort on the part of the authors to explore these other possibilities (see also my comments immediately above) before jumping directly to their favoured class of models. To some readers, it may appear that the authors were a bit hasty in making the transition. I am not suggesting that this is the case, I am sure the authors have done their job in convincing themselves that pooling models are intrinsically unable to explain the human results, but to a non-committed reader this seems like a difficult conclusion to swallow: after all, the human pattern doesn't look that counter-intuitive. Yes, there is a shrinking window, but that should not be much of a big deal. I think more is needed to convince readers that the failure of pooling models is a necessary conclusion. I should note that, from a personal point of view, I am very open to the idea that you need a grouping stage in order to explain this class of phenomena, so I am sympathetic to the conclusions of this paper. But I would still like to be convinced that this is necessary given the data, otherwise this kind of study runs into the risk of simply establishing a "widely accepted notion" that, after a few publications claiming the same, makes people lower their guard.**

Thank you for this comment. First, we would like to repeat here that our study does not merely try to explain a particular class of phenomena with different kinds of models. Instead, our study tests how different models that are known for capturing other key characteristics of crowding generalize to this paradigm. In other words, we show that grouping and segmentation processes are a natural extension of pooling models and help them capture most effects of global configuration in crowding.

Second, we implemented your suggestion of tuning parameters of pooling models to fit the human data in dense displays (preference measure; now selection measure) and checking how they behave in sparse displays (see S9 Appendix). Besides, we ran 3 control models for the capsule network (see S6 Appendix), so that the comparison to feedforward CNNs and other CNNs that do not include grouping processes is fairer.

Third, we modified the code that we shared, so that explicit instructions are provided to help other researchers to easily test their model. In the discussion, we mention other alternatives and why we think some of them cannot explain both uncrowding and crowding in dense displays.

***Minor***

**In Suppl. Material, the authors describe the preference map generated by Popart as being closer to human than Capsule Nets. I did not understand this part. When I look at the human data, I see modulations above and below the target, with little indication of any effect to the sides (left/right). This is what I see for Capsule Nets. Popart, on the other hand, presents a more isotropic pattern, which to me seems less consistent with the human pattern. Please clarify.**

Thank you for this remark. We agree that it was not clear. First, in the data of Van der Burg et al. (2019), all target closest neighbours are close to the significance level in the preference measure (now selection measure). We added an appendix figure (see S11 Appendix) in which the values of this measure are not thresholded (i.e., not set to zero if below the significance level). More importantly, as mentioned in the manuscript, the capsule networks are hard to train. Out of 10 trained networks (using the method described in the corresponding appendix), only one was able to reach 67% of performance in dense displays. We made this point clearer in the discussion. However, your remark made us realise that suggesting that a particular model does better than all others goes against the main idea of this manuscript. Indeed, our main goal was to compare different classes of models and have a general idea of which one best reproduce human behaviour, not to point towards a particular model implementation. We modified the text in discussion.

**Small suggestions:**

**- in Figure 1b: label y axis with something like "easier/harder" to clarify at a glance that small values are better**

Thank you, we added these labels to Fig 1b.

**- in the Methods section (line 161), stimulus size is described in real spatial units (deg). I understand the authors carried out some additional human measurements for this paper, so it makes sense to specify the stimulus in real-world units, but this paragraph starts by saying that humans were replaced by models. When readers reach the next 3 lines, they'll wonder what it means to define stimuli in deg for a model. So maybe add some clarification here to justify real spatial units.**

Thank you for noticing this issue. We added a clarification in the Methods section, and we specified how the different models encode spatial units in their respective appendices.
